# Simulations and error analysis of the CNC milling of a face gear tooth with given tool paths

**DOI:** 10.1016/j.dib.2019.104145

**Published:** 2019-06-12

**Authors:** Huicheng Yi, Yuansheng Zhou, Jinyuan Tang, Zezhong C. Chen

**Affiliations:** a College of Automative Engineering, Hunan Industry Polytechnic, Changsha, Hunan, 410082, China; b State Key Laboratory of High Performance Complex Manufacturing, Central South University, Changsha, 410083, China; c College of Mechanical and Electrical Engineering, Central South University, Changsha, 410083, China; d Department of Mechanical and Industrial Engineering, Concordia University, Montreal, H3G1M8, Canada

**Keywords:** CNC milling, Face gears, Simulation, Tool path, Error analysis

## Abstract

This data article gives the validation files to the article “CNC milling of face gears with a novel geometric analysis” [1]. The data is about the simulation and machining error analysis of the CNC milling of a face gear tooth with given tool paths. It includes four files. Three of them are simulation videos of the CNC milling process in VERICUT with a general view, partial view and enlarged view, respectively. The other one is the source file of the machining error analysis, and it has the design model of the face gear, the simulated machined model of the face gear, and machining error analysis according to the comparison of the design model and simulated machined model.

**Table:** Specifications Table

Subject area	Engineering
More specific subject area	Manufacturing, CNC machining
Type of data	Video, CATIA file
How data was acquired	1) Simulation in VERICUT 2) Modeling and analysis in CATIA
Data format	Raw, analyzed
Experimental factors	1) With the given tool paths, cutter and workpiece, the machining process is simulated in VERICUT. Subsequently, the simulation is recorded as videos. 2) The simulation result is output to compare with previous design model. The comparison is implemented with the distance analysis function in CATIA.
Experimental features	1) The machining process is recorded according to different views. Each view corresponds a video. 2) The result error analysis
Data source location	The data sources for the given tool path, cutters and workpiece are stated in the article [1].
Data accessibility	The data is with this article
Related article	Y. Zhou, S. Wang, L. Wang, J. Tang, Z. Chen, CNC milling of face gears with a novel geometric analysis, Mechanism and Machine Theory, 139 (2019) 46–65.

**Table:** 

Value of the data • It simulates the CNC milling process in VERICUT with given tool paths • The simulation result is compared with the design model in CATIA • The machining error analysis is implemented in CATIA

## 1 Data

This article gives the validation files for the simulation of the CNC milling of a face gear tooth. The data include three video files and one CATIA file, as stated in Table 1.

**Table 1:** Description of the given data.

File name	Contents
Simulation_GeneralView.mp4	A general view for the machining simulation in VERICUT to the generated tool paths with the proposed algorithm
Simulation_PartialView.mp4	A partial view for the machining simulation in VERICUT to the generated tool paths with the proposed algorithm
Simulation_EnlagredView.mp4	An enlarged partial view for the machining simulation in VERICUT to the generated tool paths with the proposed algorithm
Machining errors analysis.CATPart	File of simulated machining errors. It includes the following models and results: 1) the design model of face gear; 2) the simulated machined model generated from VERICUT; 3) machining error analysis according to the comparison of the design model and simulated machined model

The video files are the simulation videos of the machining process with the given tool paths, cutters, and workpiece in the commercial machining software, VERICUT. Different views are applied in different videos.

When the machining process is simulated in VERICUT, the result, a simulated machined model, can be output as a STL file, which can be input into CATIA to implement the machining error analysis. Based on this idea, the source file and analysis result are shown in the given CATIA file. Both the design model and the simulated machined model are given, and they are compared with the distance analysis function of CATIA. Subsequently, the data of the analysis result is obtained and shown in the given CATIA file.

## 2 Experimental design, materials and methods

The generation of the given data in this article is stated as the following three points.

1) The generation of the CNC milling simulation
The simulation is implemented in VERICUT, which is CNC machining simulation software. The simulation can be implemented with the given tool paths, cutters and workpiece. The tool paths are usually obtained from calculations [1], or generated from other commercial CNC machining software. The workpiece can either imported or defined in VERICUT. Usually, if the workpiece has a simple geometry, such as the data ‘Simulation_GeneralView.mp4’, it is directly defined in VERICUT. If the workpiece has a complex geometry, such as the data ‘Simulation_PartialView.mp4’ or ‘Simulation_EnlagredView.mp4’, it is imported from the existed model, which is modeled in commercial CAD software or imported from of previous simulation.
2) The generation of 3D design model of face gears
With the design parameters of a face gear drive, the tooth surface points can be calculated in an even distribution on the tooth surface. Subsequently, the tooth surface points can be imported into commercial CAD software to be connected as curves and surface. With the tooth surface model, the 3D model can be easily obtained in CAD software. The similar process and the details can be referred to Refs. [1], [2], [3], [4].
3) The machining error analysis
The machining error analysis is implemented in CATIA as the comparison result of the design model and simulated machined model. The design model is built in CATIA based on the tooth surface points calculation according to the given design parameters [1]. The simulated machined model is imported from the result of the VERICUT simulation.
